# Integrated analysis of lncRNA-mediated ceRNA network involved in immune regulation in the spleen of Meishan piglets

**DOI:** 10.3389/fvets.2022.1031786

**Published:** 2022-10-19

**Authors:** Jing Shi, Chao Xu, Zhengchang Wu, Wenbin Bao, Shenglong Wu

**Affiliations:** ^1^Key Laboratory for Animal Genetics, Breeding, Reproduction and Molecular Design of Jiangsu Province, College of Animal Science and Technology, Yangzhou University, Yangzhou, China; ^2^Joint International Research Laboratory of Agriculture and Agri-Product Safety, Yangzhou University, Yangzhou, China

**Keywords:** Meishan piglet, spleen, mRNA, lncRNA, WGCNA, ceRNA

## Abstract

Meishan pigs are a famous local pig breed in China, with high fertility and early sexual maturity, and stronger immunity compared to other breeds. The spleen is the largest lymphoid organ in pigs and performs essential functions, such as those relating to immunity and haematopoiesis. The invasion of many pathogenic microorganisms in pigs is associated with spleen damage. Long non-coding RNAs participate in a broad range of biological processes and have been demonstrated to be associated with splenic immune regulation. However, the expression network of mRNAs and lncRNAs in the spleen of Meishan pigs remains unclear. This study collected spleen tissues from Meishan piglets at three different ages as a model, and mRNA and lncRNA transcripts were profiled for each sample. Additionally, 1,806 differential mRNAs and 319 differential lncRNAs were identified. A complicated interaction between mRNAs and lncRNAs was identified *via* WGCNA, demonstrating that lncRNAs are a crucial regulatory component in mRNA. The results show that the modules black and red have similar mRNA and lncRNA transcription patterns and are mainly involved in the process of the immune defense response. The core genes (DHX58 and IFIT1) and key lncRNAs (TCONS-00002102 and TCONS-00012474) of piglet spleen tissue were screened using the ceRNA network. The expression of these genes is related to the immune response of pigs. Our research may contribute to a further understanding of mRNA and lncRNA expression in the spleen of piglets, and provide new ideas to improve the disease resistance of piglets.

## Introduction

With the rapid development of high-throughput, transcriptome-sequencing technologies, our understanding of the mammalian genome is growing rapidly. This has contributed to the improvement of economic traits in mammals, such as beef cattle (1) and Ningxiang pigs (2). Although approximately two-thirds of the mammalian genome is actively transcribed, most does not encode proteins (3). Long non-coding RNA (lncRNA) is a type of non-coding RNA that accounts for more than 80% of non-coding RNAs (4), with fragments larger than 200 bp, mostly without coding ability (very few can encode small peptides), and is directly involved in the regulation of expression in cells (5). A series of recent papers have shown that cis-lncRNA and trans-lncRNA play a role in regulating the activity of genes (6). Moreover, lncRNA can act as a positive or negative signal during transcription, regulate protein activity, and modulate chromatin function (7–9). Numerous studies have also shown that lncRNAs play an essential regulatory role in immune-related processes in pigs, such as physiopathology. For example, Wu et al. identified differentially expressed lncRNAs and mRNAs in porcine alveolar macrophages after infection with PRRSV (Porcine Reproductive and Respiratory Syndrome Virus) and found that co-expressed genes for down-regulated lncRNAs were significantly enriched in NF-κB and Toll-like receptor signaling pathways (10). Chen et al. identified the expression profile of lncRNAs in IPEC-J2 cells during PEDV (Porcine Endemic Diarrhea Virus) infection and further determined the differential expression of immune-related lncRNAs in PEDV-infected IPEC-J2 cells and newborn piglets (11). Fang et al. analyzed 199 differentially expressed lncRNAs in IPEC-J2 cells after PCV2 (Porcine Circovirus Type 2) infection, and their regulatory target genes (SOD2, TNFAIP3, and MG7) were all associated with infectious diseases (12). LncRNAs, especially during transcription, have remained a research hotspot in recent years. Therefore, in the present study, we investigated the mRNA regulation of lncRNAs in Meishan piglets.

China has approximately 100 breeds of domestic pig genetic resources, accounting for over one-third of the global total (13). The Meishan pig is a famous local pig in China, known for its high fecundity and early sexual maturity. Additionally, many domestic and foreign studies have shown that Meishan pigs have shown stronger tolerance and resistance to many diseases. For example, Halbur et al. (14) found that Meishan pigs were less susceptible to Porcine Reproductive and Respiratory Syndrome Virus (PRRSV) than Hampshire and Duroc pigs. Reiner et al. (15) found that Meishan pigs were more resistant to pork sporozoites than Pietrain pigs. In addition, Chen et al. (16) compared the expression of porcine β-defensins in Meishan pigs and crossbred (Duroc × Yorkshire × Landrace) pigs and found that Meishan pigs had a higher expression, which might be the reason for their higher immunity and disease resistance. Dong et al. (17) compared the intestinal barrier function of Meishan piglets and crossbred neonatal piglets and found that Meishan pigs had greater intestinal barrier function. Therefore, it is of great interest to study the immune resistance of Meishan pigs.

Pig farming is a pillar of China's livestock industry, with epidemic problems causing considerable losses to the pig industry every year. In particular, piglets are less resistant to disease than adult pigs, and there are differences between breeds in the resistance of pigs to disease (18). PED (Porcine Epidemic Diarrhea) affects pigs of all ages, but lethality is seen mainly in lactating piglets (19). Newborn piglets infected with PDCoV (Porcine Delta Coronavirus) die from severe diarrhea (20). Infection with TGEV (Transmissible Gastroenteritis Virus) causes 100% mortality in piglets under 14 days of age (21). It is therefore a matter of urgency to pay attention to and improve piglets' immunity and disease resistance. As the most prominent secondary lymphoid organ in pigs, the spleen contains a variety of immunoreactive cells and immune factors. It is an important site of response for both innate and adaptive immunity. In recent years, many studies have been conducted to screen for immune-related genes in pigs by the transcriptome analysis of porcine immune organs in order to improve resistance and resilience to pathogens (22–25).

However, no RNA sequencing study has yet been reported for Meishan pigs. In order to investigate the molecular regulatory mechanisms of immune differences among Meishan piglets, the spleen was used as an immune model. The spleens were collected from Meishan piglets at three ages: 1 day old (without colostrum), 14 days after colostrum feeding, and 28 days after colostrum feeding. Additionally, the mRNA and lncRNA sequencing of spleen tissues was performed by high-throughput sequencing technology. We successfully identified differentially expressed candidate genes, thus providing a reference for studying the function and mechanism of lncRNAs in spleen tissues. In addition, our study will increase our understanding of the transcriptomics associated with spleen tissue and contribute to a better understanding of the immune function of the mammalian spleen.

## Materials and methods

### Ethics statement

All experiments were approved by the Institutional Animal Care and Use Committee (IACUC) of Yangzhou University (Pig: SYXK(Su)2012-0029) and were performed according to the Animal Ethics Procedures and Guidelines of the People's Republic of China. No other specific permissions were required for these experiments.

### Experimental animal and sample collection

Two Meishan sows were chosen that were similar in weight, age, and body shape and that had farrowed on the same day. After parturition, the piglets received the same diet and were housed in an environmentally controlled room. We designated the first day of the newborns' existence as day 1. Once the sows farrowed, two piglets from each litter were immediately chosen to be slaughtered. Four piglets of similar weight were sacrificed. Following slaughter, spleen samples were collected simultaneously and snap-frozen in liquid nitrogen (-196°C). The remaining piglets were housed in two pens in an environmentally controlled room and were fed under identical husbandry conditions. All piglets were fed only *via* breastfeeding until weaning (day 35). Similar to the aforementioned sample collection procedure on day 1, two piglets from each litter were chosen to be sacrificed at postnatal days 14 and 28 after farrowing using an intravenous injection of pentobarbital sodium, which minimized animal suffering. Spleen samples were immediately stored in liquid nitrogen for RNA isolation. Samples were kept at ultra-low temperatures to avoid RNA degradation.

### RNA extraction

Total RNA from the milled spleen tissues was extracted using Trizol reagent (TaKaRa, Dalian, China), according to the manufacturer's protocol. The extracted total RNA was then treated with RNase-free DNase to remove excess DNA. The quality of the RNA extracted from spleen tissue was assessed by Nanodrop 2000 (Thermo Fisher Scientific, Waltham, MA, USA). Qualified total RNA was stored at −80°C until use. A total of 12 samples were used for RNA extraction.

### Library construction and RNA-Seq analysis

Qualified RNA samples of four individuals at the same age were equally pooled together to form three RNA groups: 1 d, 14 d, and 28 d. Approximately 1 μg of total RNA per sample was treated with the Ribo-Zero™ Magnetic Kit (Epicenter) to deplete rRNA. The retrieved RNA was fragmented by adding First Strand Master Mix (Invitrogen). First-strand cDNA was generated using random primer reverse transcription, followed by second-strand cDNA synthesis. The synthesized cDNA was subjected to end-repair and then was 3' adenylated. Adapters were ligated to the ends of these 3' adenylated cDNA fragments. Several rounds of PCR amplification with PCR Primer Cocktail and PCR Master Mix were performed to enrich the cDNA fragments. Then, the PCR products were purified with Ampure XP Beads. The constructed RNA libraries were quality checked with an Agilent 2100 Bioanalyzer and then sequenced using an Illumina sequencer.

### Raw data quality assessment

Raw sequencing data (Raw Reads) were first filtered to obtain high-quality clean data to ensure the quality and accuracy of subsequent bioinformatic analysis. The quality control of the raw and trimmed reads was performed using FastQC and MultiQC (26, 27). Trimming of the adapter content and quality trimming was performed using Cutadapt (28). All downstream analyses were based on high-quality clean reads.

### Identification and classification of lncRNAs

Clean reads aligned to the reference genome (Sus scrofa 11.1) were stored in a binary bam file. The new transcript was spliced after the readings were collated using Stringtie software (29). Then, by comparing the gene annotation data of the reference sequence generated by Cuffcompare software (30), the potential lncRNA transcripts were chosen. CPC, CNCI, Pfam, and PLEK were used to filter out lncRNAs with coding potential and obtain the predicted lncRNA sequences (31–34).

### Functional enrichment and differential expression analysis

Using the R package DESeq2 (35), differential expression gene analysis was conducted between the two groups, and genes with p-adj <0.05 and |log2FoldChange| > 1 were chosen as differential genes. By performing a hypergeometric distribution test using enrichGO and enrichKEGG in the R package clusterProfiler (36), the functional enrichment analysis of genes based on the GO and KEGG databases was carried out and enriched pathways with *p* < 0.05 were retained.

### Co-expression networks (weighted correlation network analysis)

Weighted gene co-expression network analysis (WGCNA) can be used to build gene co-expression modules using gene expression profiles (37, 38). The gene relationship matrix was first derived from the gene expression matrix using the Pearson correlation coefficient. By setting a soft threshold of 9, the gene relationship matrix was transformed into an adjacency matrix. The network's interconnectivity was then determined using the topological overlap matrix (TOM). In order to categorize genes into various modules, we employed the TOM difference degree as the clustering distance. Using a threshold of 0.25, the dynamic tree approach was also employed to combine related gene modules (37).

### Construction of mRNA–lncRNA and mRNA–lncRNA–pathway networks

To explore the association between mRNA and lncRNA using the significant module of the mRNA–lncRNA co-expression network, we constructed mRNA–lncRNA–pathway networks based on mRNA–lncRNA networks and important pathways involved in mRNA regulation. This paper aims to reveal the relevant pathways of lncRNA regulation and thus predict the possible mechanisms of lncRNA in the spleen. The network was constructed using significant correlation pairs based on Pearson correlation coefficients. The differential co-expression network was visualized and analyzed using Cytoscape software (version: 3.9.0) (39).

### Establishment of the lncRNA–miRNA–mRNA ceRNA network

In 2011, Leonardo Salmena proposed the competing endogenous RNA (ceRNA) hypothesis, revealing a new mechanism of RNA interactions. This hypothesis suggested that different types of RNA molecules competitively bind to miRNAs, thus reducing the inhibitory effect of miRNAs on their target mRNAs; these competitive endogenous RNAs may include circRNAs, lncRNAs, pseudogenes, and protein-coding mRNAs (40). To better understand the role of lncRNAs in the ceRNA network, all potential co-deregulated competitive triads were established to construct the lncRNA–miRNA–mRNA network. The miRNA sequences were downloaded from the miRBase website. The MiRanda software (41) was used to predict miRNA-targeted lncRNAs and mRNAs. A max score > 190 and max energy <−15 were set to obtain high confidence in the interaction relationships. The ceRNA network was then constructed from co-expressed mRNA–lncRNAs and their co-targeted miRNAs (42).

### Real-time PCR quantification

Total RNAs from pig spleen tissues were extracted using Trizol (TaKaRa, Dalian, China). Then, reverse transcription of RNA was conducted using HiScript III RT SuperMix with gDNA wiper (Vazyme, Nanjing, China). The RT-qPCR reaction system contained 5 μl SYBR Green Mixture (Vazyme, Nanjing, China), 1 μl of the cDNA template, 0.2 μl of each primer, and 3.6 μl deionised water. The thermal conditions were as follows: 95°C for 5 min, 40 cycles of 95°C for 10 s, and 60°C for 30 s. The GAPDH genes were set as the internal controls. All the forward and reverse primers for the RT-qPCR assays are listed in Table 1. The expression level of each validated gene for each timepoint was calculated by the 2^−Δ*ΔCt*^ method.

**Table 1:** Forward and reverse primers used for gene quantification by RT-qPCR.

**Name**	**Sequence (5'−3')**
IFIT1-F	CTTGGAGGAGATTGAGTT
IFIT1-R	CAGTATGTTCTTGTTGGG
DHX58-F	CTCTGTGCCAACCT
DHX58-R	TCCCGTCTCAACTC
TCONS-00012474-F	GAGCCACAAAGGGAA
TCONS-00012474-R	GCTGAGGTGAGGTAA
TCONS-00002102-F	GCCCTTCTACCCTATCAT
TCONS-00002102-R	ATTTCCTTTCACCGACTC

## Results

### Identification and classification of lncRNAs in the spleen tissue of Meishan piglets

**Figure 1:**
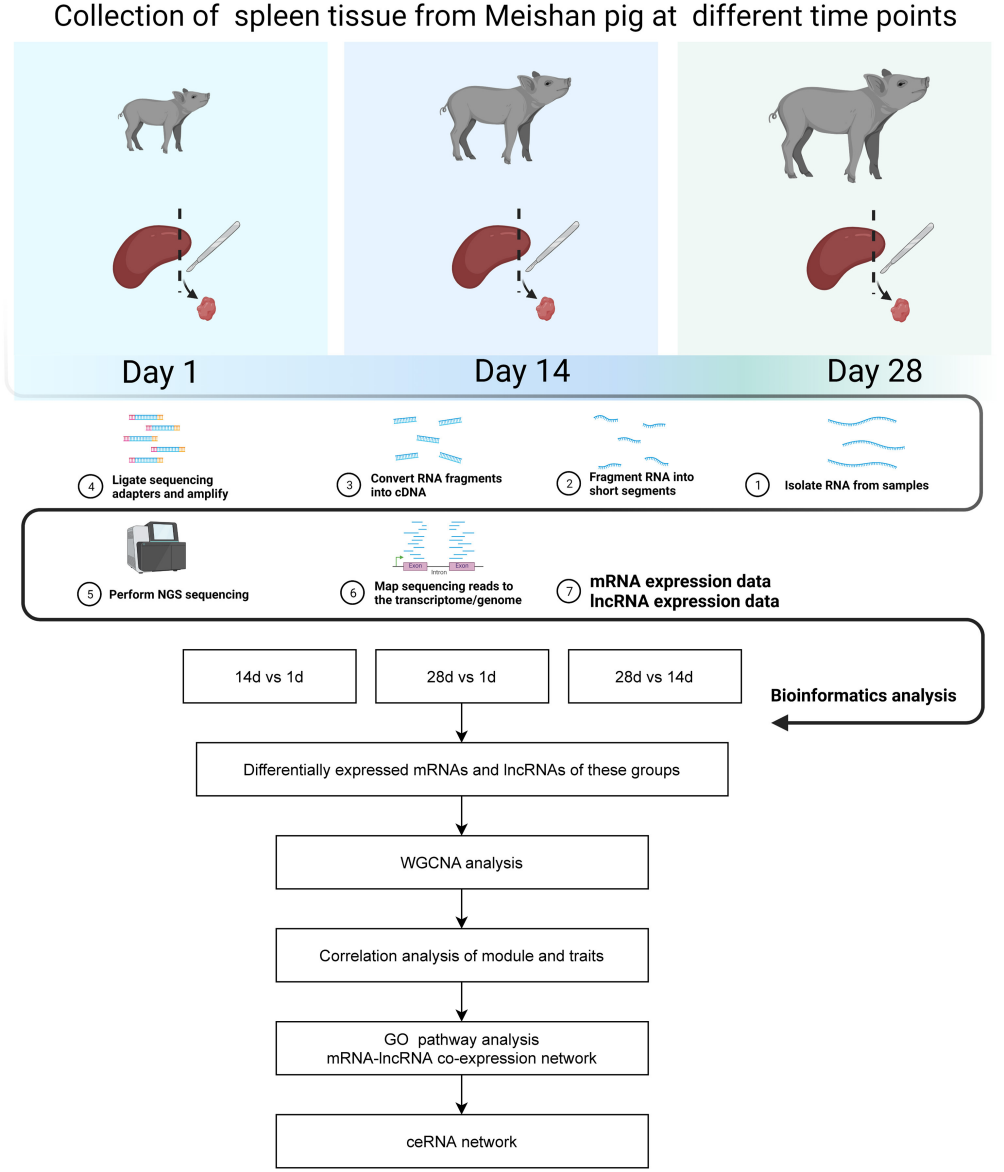
Flow chart of data analysis.

**Figure 2:**
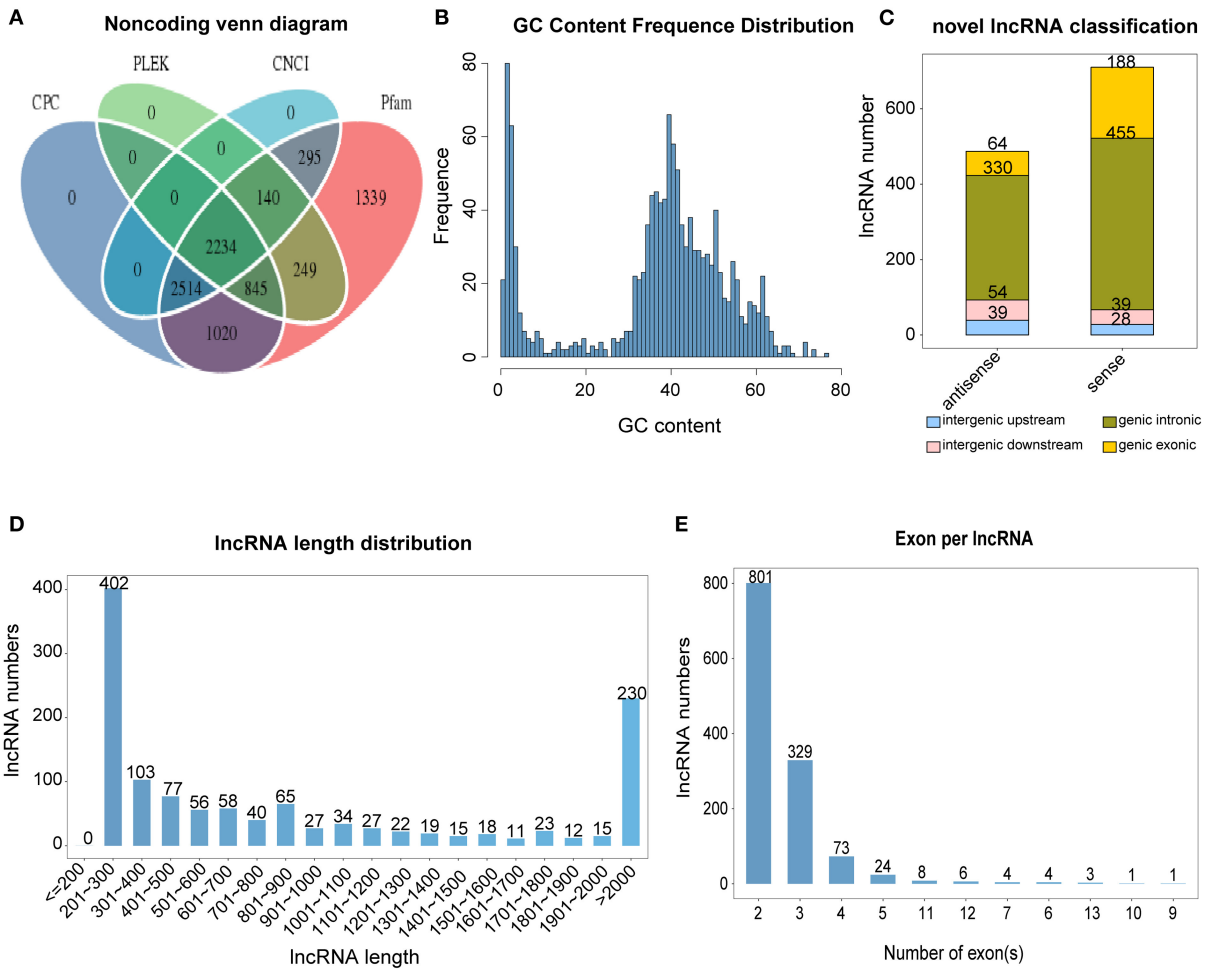
Classification and identification of lncRNAs. **(A)** Venn diagram of lncRNA, predicted using CPC (Coding Potential Calculator), CNCI (Coding-Non-Coding Index), CPAT (Coding Potential Assessment Tool), and Pfam (Protein families). **(B)** lncRNA GC content. **(C)** lncRNA classification. **(D)** lncRNA length. **(E)** lncRNA exon number.

The data analysis process for this study is shown in Figure 1. We used a combination of the most widely used coding potential analysis methods to screen the candidate lncRNAs, including CPC analysis, CNCI analysis, Pfam protein structural domain analysis, and PLEK analysis. The resulting Venn diagram shows that a total of 2,234 new lncRNA transcripts were detected using the four methods (Figure 2A). The statistical distribution of the GC content of the predicted lncRNA sequences showed that most of the lncRNA GC content was around 30–60% (Figure 2B). The stacked bar chart shows that the highest proportion of newly predicted lncRNAs was genic intronic in both the antisense and sense categories (Figure 2C). Figure 2D shows the length distribution of lncRNAs, with more lncRNAs being between 200 and 300 bp in length (Figure 2D). The results of the exon number distribution map of lncRNAs show that lncRNAs were mainly concentrated in two exons, followed by three exons (Figure 2E).

### Analysis of differentially expressed mRNA and lncRNA

**Figure 3:**
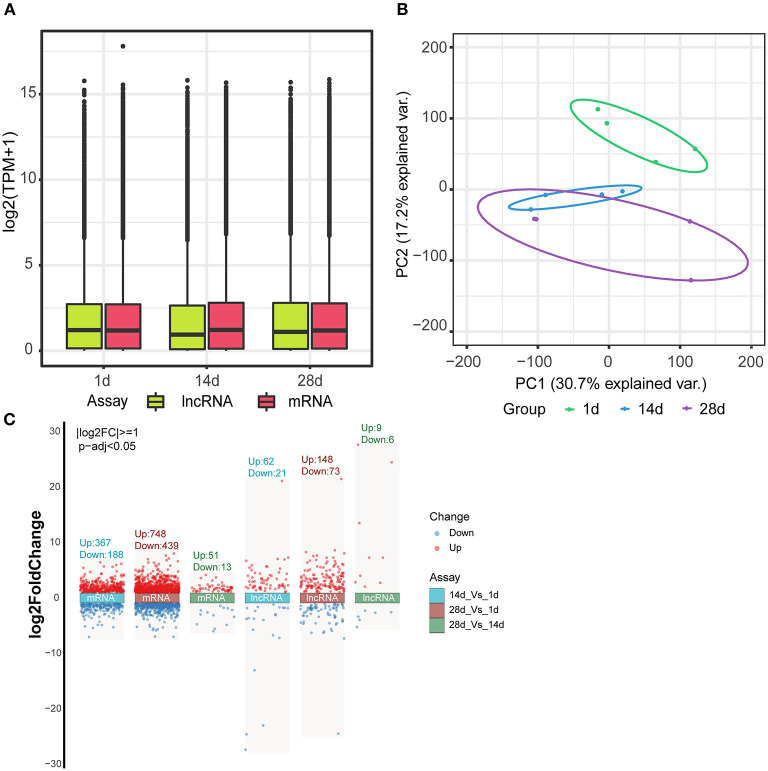
Differential expression of mRNAs and lncRNAs. **(A)** Box line plot of mRNA and lncRNA expression in three different groups of spleen tissues. **(B)** Principal component analysis of mRNA and lncRNA in three different groups of spleen tissues. **(C)** Volcano plot analysis of mRNA and lncRNA expression between the three groups.

The box plot demonstrates the consistency in the distribution of mRNA and lncRNA in terms of the transcript expression levels across the three groups, indicating no batch effect in the data (Figure 3A). Principal component analysis showed that the three different groups of spleens essentially formed well-defined groups, ranked according to their number of days (Figure 3B). Day 1 and days 14 and 28 clustered more distantly, while days 14 and 28 clustered more closely with each other. Differences in the expression of mRNAs and lncRNAs between the three groups were analyzed by volcano plots, which showed 367 up-regulated genes and 188 down-regulated genes for 14 vs. 1 d, 748 up-regulated genes and 439 down-regulated genes for 28 vs. 1 d, and 51 up-regulated genes and 13 down-regulated genes for 28 vs. 14 d in mRNAs. In terms of lncRNAs, 14 vs. 1 d had 62 up-regulated genes and 21 down-regulated genes, 28 vs. 1 d had 148 up-regulated genes and 73 down-regulated genes, and 28 vs. 14 d had 9 up-regulated genes and 6 down-regulated genes (Figure 3C, Supplementary Tables 1, 2).

### GO and KEGG enrichment analysis of differentially expressed mRNAs

**Figure 4:**
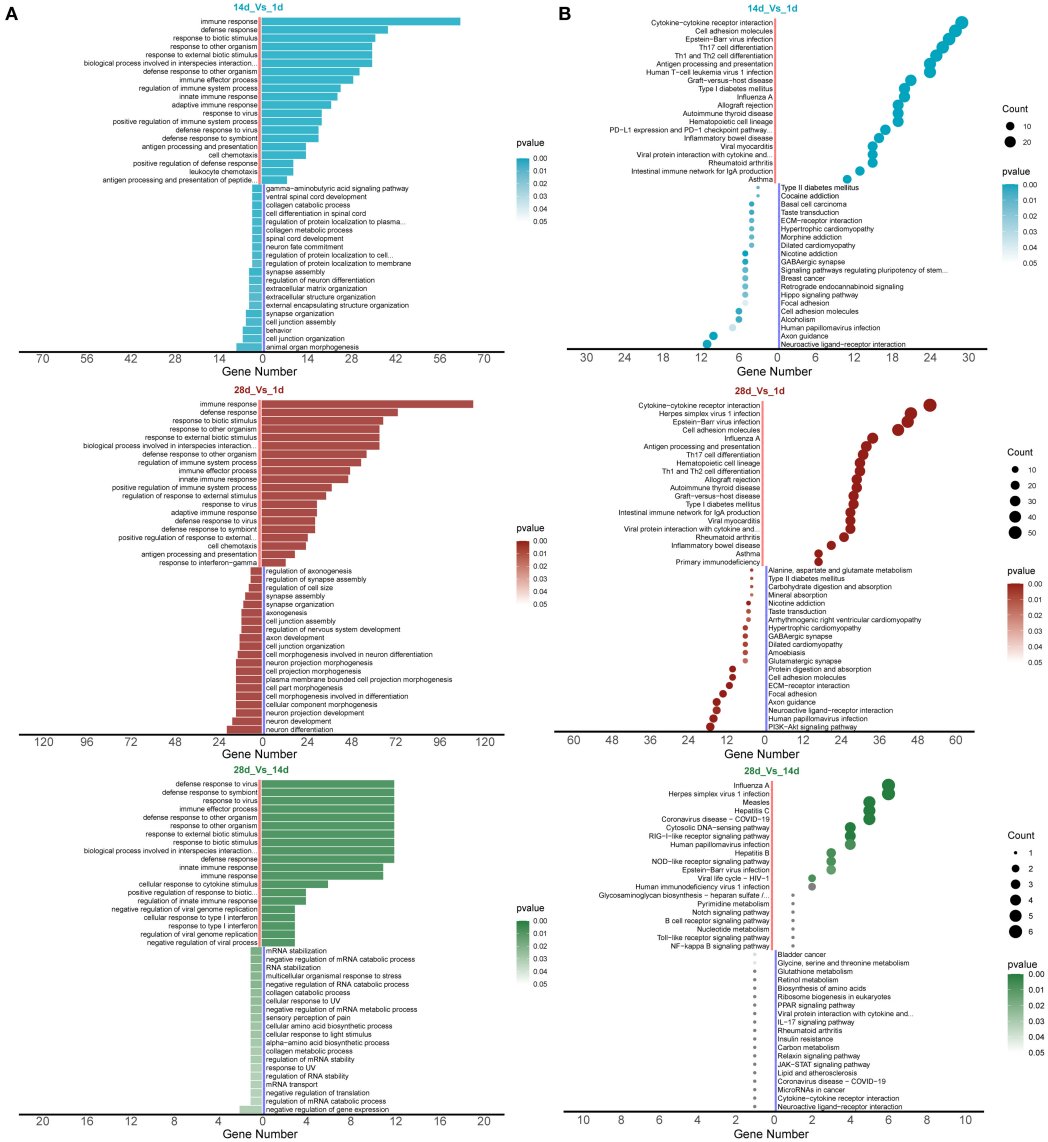
mRNA enrichment analysis for differential expression. **(A)** GO enrichment analysis and **(B)** KEGG enrichment analysis.

GO analysis revealed that the biological processes and pathways enriched by the differentially expressed mRNAs in piglet spleen tissues were mainly related to the immune response, defense response, and defense response to a virus (Figure 4A, Supplementary Tables 3–5). KEGG pathway enrichment analysis showed that cytokine–cytokine receptor interaction, haematopoietic cell lineage, toll-like receptor signaling pathway, and the NF-kappa B signaling pathway were the most important pathways (Figure 4B, Supplementary Tables 6–8).

### WGCNA analysis

**Figure 5:**
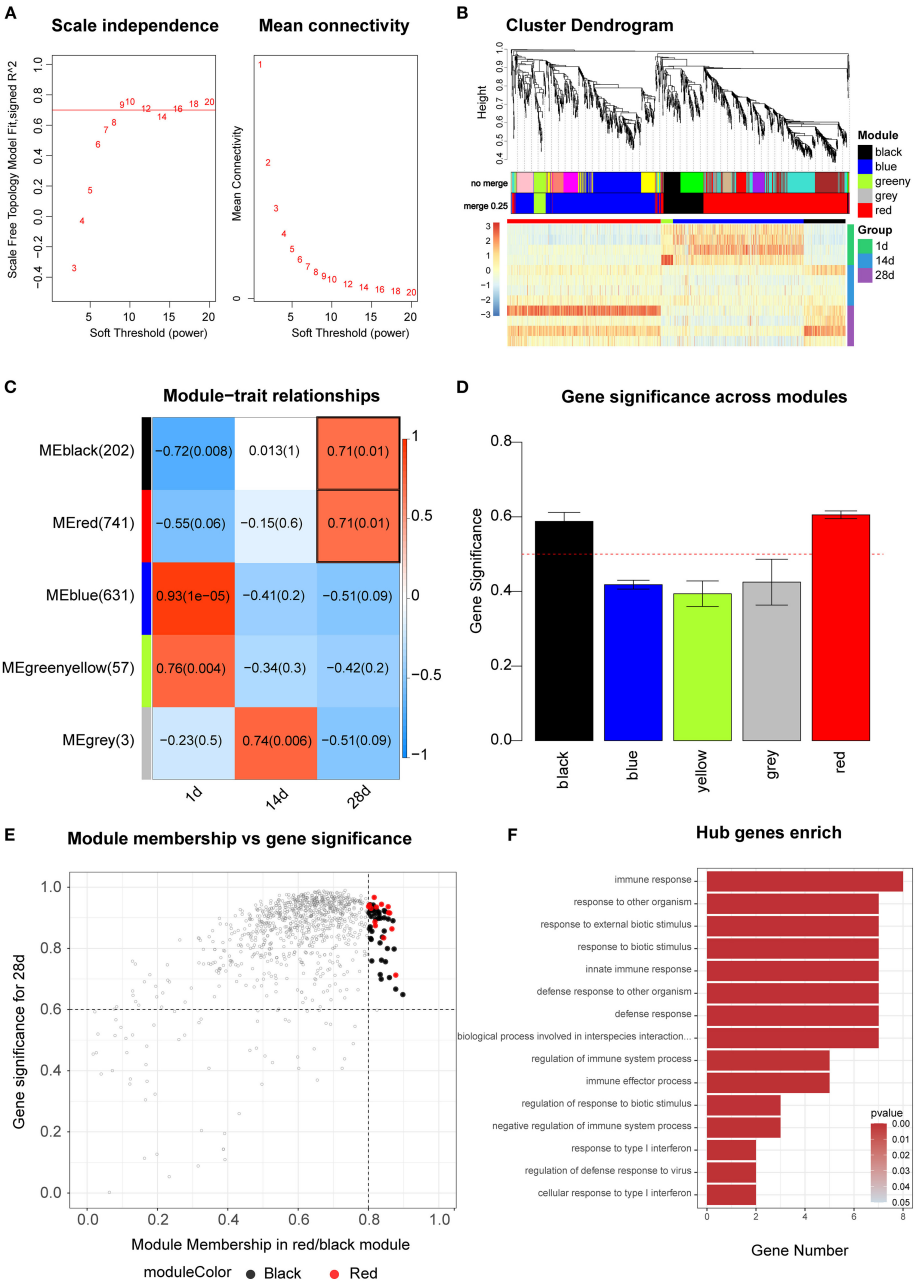
WGCNA analysis. **(A)** Scale independence and mean connectivity analysis for various soft threshold powers. **(B)** Clustering dendrograms of mRNAs. Different colors indicate different co-expression modules. Heat map showing the expression profile of protein-coding genes. **(C)** Module–trait relationship. Each row represents a module eigengene, and each column represents a trait. Each cell includes the corresponding correlation and *p*-value. **(D)** Gene significance analysis for each of the 28 d modules. **(E)** Scatter plot of red and black modules. **(F)** GO enrichment analysis of the pathways of the red and black modules.

WGCNA was used to construct a differentially expressed co-expression module comprising 1,806 mRNAs and 319 lncRNAs. When the soft threshold power β was set at 9, the scale-free network matching index exceeded 0.7, which is of greater biological significance (Figure 5A). Therefore, β = 9 was used to generate a hierarchical clustering tree. A co-expression network (combined cut height = 0.25, redundancy = 3) was constructed by WGCNA to discover the potential regulatory functional relationships between lncRNAs and mRNAs in spleen tissues at different ages, as well as their mechanisms. The network was divided into five modules, identified by and displayed in different colors (black, blue, yellow, gray, and red), each containing different gene clusters and showing the expression patterns of genes within the different modules in a heat map (Figure 5B, Supplementary Table 9). Red represents positive correlations, while blue represents negative correlations. The relationship between co-expression modules and different ages is shown in Figure 5C. We found a significant positive correlation between the black and red modules and 28 days (correlation coefficient for 28 d = 0.71, *p* = 0.01). Gene significance analysis was performed for each of the 28 d modules (Figure 5D), and the red and black modules were significant > 0.5; thus, the red and black modules for 28 d were selected for the next step of the analysis. Figure 5E shows the significance of these genes in the red and black module for 28 d. Furthermore, genes with gene significance > 0.6 and module membership > 0.8 were used as core genes. There were 69 mRNAs and 24 lncRNAs in the black and red modules (Figure 5E). Performing GO enrichment analysis of mRNAs, we found that the red and black modules are mainly involved in the process of the immune defense response (Figure 5F, Supplementary Table 10).

### Construction of mRNA–lncRNA co-expression networks and ceRNA networks

**Figure 6:**
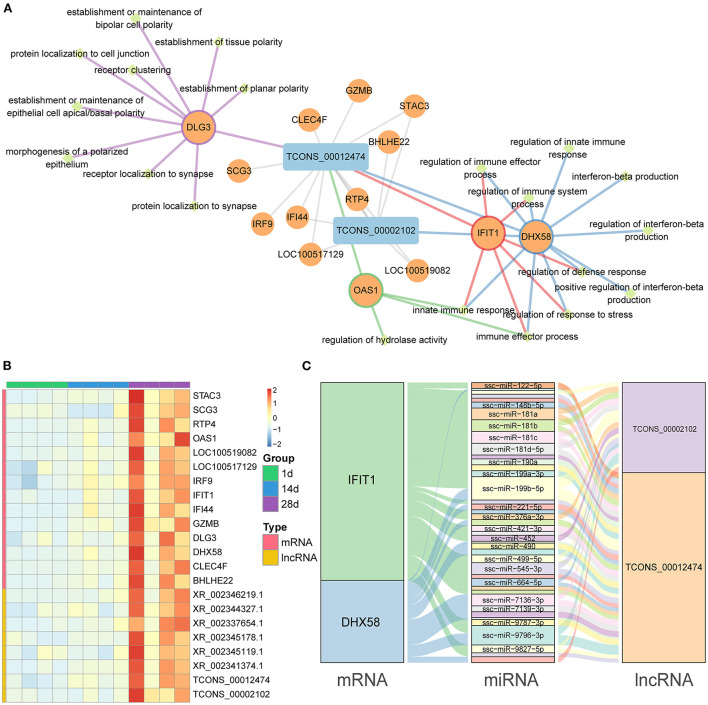
mRNA–lncRNA co-expression networks and ceRNA networks. **(A)** Co-expression mRNA–lncRNA network in the red and black modules. The circular nodes represent the mRNAs, and the triangle nodes represent lncRNAs. Gray edges represent mRNA–lncRNA interactions and the other edges represent the mRNA pathways. **(B)** Heat map showing the correlated expression of mRNA and lncRNA in spleen tissue at three different ages. **(C)** ceRNA network shows the relationship between mRNA, miRNA, and lncRNA.

In our study, we identified 14 mRNAs and 2 lncRNAs in the mRNA–lncRNA pathway network, all of which were upregulated at 28 days (Figure 6A). In the mRNA–lncRNA network, *TCONS-00012474* (one lncRNA) was linked to 14 mRNAs (*DLG3, SCG3, CLEC4F, GZMB, IRF9, IFI44, RTP4, BHLHE22, STAC3, LOC100517129, OAS1,LOC100519082, IFIT1*, and *DHX58*), corresponding to the linkage and enrichment of the innate immune response, immune effector process, and other pathways. *TCONS-00002102* (one lncRNA) is related to seven mRNAs (*IF144, RTP4, BHLHE22, STAC3, LOC100517129, LOC100519082*, and *DHX58*) and is enriched in the innate immune response, immune effector process, and other pathways. A heat map of the topological overlap of interacting mRNAs and lncRNAs in the mRNA–lncRNA–pathway co-expression network in three age groups was created with different color markers; red represents positive correlations, while blue represents negative correlations (Figure 6B). We predicted miRNAs common to mRNA and lncRNA to construct the mRNA–miRNA–lncRNA ceRNA network (Figure 6C, Supplementary Table 11), which included 2 lncRNAs, 2 mRNAs, and 41 miRNAs.

### RT-qPCR quantification of mRNAs and lncRNAs

**Figure 7:**
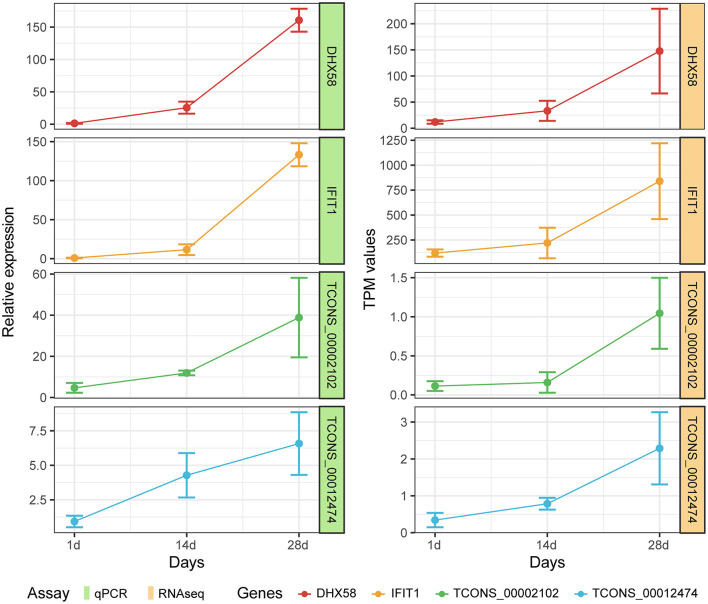
Expression patterns of DHX58, IFIT1, TCONS-00002102, and TCONS-00012474 compared with expression patterns obtained by RNA-seq.

In order to confirm the reliability of RNA-seq data in Meishan piglets, the mRNA (*IFIT1* and *DHX58*) and lncRNA (*TCONS-00002102* and *TCONS-00012474*) genes were selected for RT-qPCR. It can be seen that the RT-qPCR results for these mRNAs and lncRNAs are similar to the RNA-seq results, indicating the accuracy of the RNA-seq data (Figure 7).

## Discussion

The spleen is one of the pigs' most important immune organs. Additionally, studies have reported many epidemics in pigs to be associated with spleen damage, such as PRRS, Porcine Circovirus, pseudorabies, and swine fever (43). There are two existing studies on lncRNAs in the spleen of Chinese endemic pigs, and these studies focused on the multiple developmental stages of the pig spleen (22, 43). In our study, Meishan piglets were selected as a model to analyse the transcriptional expression of lncRNA and mRNA in the spleen tissue of piglets for the first time. The differentially expressed 1,806 mRNAs and 319 lncRNAs were identified based on transcriptome expression profiles.

The colostrum is considered to be the first immunization for newborns. Therefore, the three different sample groups selected in this study exhibited large immunological differences. Compared to 14 vs. 1 d and 28 vs. 1 d, the number of differential mRNAs and lncRNAs in 28 vs. 14 d is significantly lower. We speculate that the immune system of piglets starts to establish itself before 14 days, which is in general agreement with the view that the immune system of pigs is established at seven days of life (43).

The results of GO enrichment analysis showed an increase in the expression of genes in pathways related to the immune defense response to viruses. The changes reflect the immunologic function of the spleen (44). In addition, the KEGG results were enriched for upregulating the haematopoietic cell lineage pathway. This result suggests that the spleen may be involved in haematopoietic processes during development (44).

The initial innate immune responses are the first line of defense against viral vectors and help modulate subsequent adaptive immune responses (45). The gene enrichment in the innate immune response and immune effector process pathways discovered by WGCNA analysis demonstrates that the innate immune function of the spleen has been activated. Interferon beta is an important type I interferon that plays an important role in intrinsic antiviral immunity (46), and has anti-tumor, anti-proliferative, and immunomodulatory functions (47, 48). The enrichment of the interferon beta production pathway indicates that the spleen of piglets already has immunomodulatory functions at 28 days of age.

*IFIT1* and *DHX58* were identified as the central genes by mRNA–lncRNA co-expression network analysis. The target genes of the two lncRNAs (*TCONS-00002102* and *TCONS-00012474*) were shown to be *IFIT1* and *DHX58* based on the predicted ceRNA network map. All genes were significantly upregulated with age. *IFIT1*, also known as *ISG56*, is a member of the family of interferon-inducible proteins with tetrapeptide repeats (IFITs) (49). IFITs are important viral restriction factors that have been shown to directly inhibit viral protein synthesis and regulate innate immune signaling pathways. Recently, it was shown that the knockdown of the STAT1 gene, a gene that inhibits Porcine Delta Coronavirus, resulted in a significant increase in PDCoV production, and then downstream interferon-stimulated gene expression was detected, in which IFIT1 expression was found to be substantially decreased (50). This suggests that IFIT1 is important in antiviral replication. Furthermore, Vaishali Sah et al. used IFIT1 as a key indicator of immunity by measuring its expression level after swine fever vaccination (51). It has been reported that interferon can significantly trigger the production of many interferon-induced genes (IFIT1, IFITM3, MX-1, OASL, ISG15, PKR, GBP1, Viperin, BST2, IRF-1, and CXCL10), which play key roles in the resistance to viral infection (52). Bo Yang et al. also showed that the expression of some antiviral and inflammation-related factors was significantly altered after African swine fever virus infection, including the interferon-inducible protein IFIT1 (53). This suggests that IFIT1 plays an important immune function in the host as an interferon-inducible gene. According to our results, IFIT1 also plays a key role in the establishment of splenic immunity in Meishan piglets. *DHX58* is a member of the retinoic-acid-inducible gene (RIG)-like receptor (RLRs) family, which are pattern recognition receptors (PRRs) that trigger an innate immune response against viral infections (54). Previous studies have shown that the mRNA and protein levels of *DHX58* are significantly upregulated in M1 macrophages (55). Moreover, the gene encoding this protein can stimulate macrophages to generate signals that incite the mitochondria to produce inflammasomes, producing inflammatory proteins that play a role in the defense response (56). In addition, it has been shown that *DHX58* negatively regulates the RIG-1 signaling pathway through the competitive binding of viral RNA molecules to RIG-I/MDA5 and inhibits the transcription of type I IFN induced by viral infection (57). Li et al. found that an SNP in the *DHX58* gene was significantly associated with blood parameters in pigs (58). With these results, we speculate that *DHX58* may be one of the crucial genes associated with the immune response in pigs.

A shortcoming of this study is that only mRNA and lncRNA transcripts were analyzed in the spleen of Meishan piglets, and the immune effects of the selected lncRNA transcripts were not explored in depth. This represents a direction for future work.

## Conclusion

In summary, the ceRNA networks were constructed by predicting miRNA-targeted lncRNAs and mRNAs, and were screened for the core genes (*DHX58, IFIT1*) and key lncRNAs (*TCONS-00002102, TCONS-00012474*). Additionally, they play a key role in immune defense, the inflammatory response, and other processes. The results of this study contribute to our understanding of the immune function of the spleen in Meishan piglets, lay the foundation for the study of lncRNAs in Meishan pigs, and provide new insights into the function of lncRNAs in spleen tissue. However, this study still has limitations, and more experiments are needed to explore the biological functions of key lncRNAs in order to improve disease resistance in piglets.

## Data availability statement

The datasets presented in this study can be found in online repositories. The names of the repository/repositories and accession number(s) can be found in the article/Supplementary material.

## Ethics statement

The animal study was reviewed and approved by Institutional Animal Care and Use Committee (IACUC) of Yangzhou University (Pig: SYXK(Su)2012-0029).

## Author contributions

JS, CX, and WB designed the experiments. JS and CX collected the experimental tissues, analyzed the data, and interpreted the results. JS wrote the manuscript with input from all the authors. SW and ZW participated in designing the structure of the article. All authors have read and approved the final manuscript.

## Funding

This work was supported by grants from the Key Research and Development Project (Modern Agriculture) of Jiangsu Province (BE2019341), the Open Competition Mechanism to Select the Best Candidates for the Foundation for Breeding Industry Prosperity of Jiangsu Province, China (JBGS[2021]098), and the Priority Academic Program Development of Jiangsu Higher Education Institutions.

## Conflict of interest

The authors declare that the research was conducted in the absence of any commercial or financial relationships that could be construed as a potential conflict of interest.

## Publisher's note

All claims expressed in this article are solely those of the authors and do not necessarily represent those of their affiliated organizations, or those of the publisher, the editors and the reviewers. Any product that may be evaluated in this article, or claim that may be made by its manufacturer, is not guaranteed or endorsed by the publisher.
